# ADHD: Beyond Core Symptoms and Specialist Care

**DOI:** 10.3390/jcm15176570

**Published:** 2026-08-26

**Authors:** Rafał R. Jaeschke

**Affiliations:** Section of Biological and Community Psychiatry, Department of Psychiatry, Jagiellonian University Medical College, 31-501 Kraków, Poland; rafal.jaeschke@gmail.com

Attention-deficit/hyperactivity disorder (ADHD) is still too often regarded as a marginal or highly specialist concern, rather than as a condition with direct relevance to everyday clinical practice [1]. This misconception has consequences at both the individual and public health levels. It adds to the lifelong suffering of many patients with ADHD and contributes to substantial health-related and occupational burden [2,3]. Unless this often-overlooked contributor to disability and ill health is recognized and addressed, efforts to optimize treatment may fall short of what patients need.

The clinical burden of ADHD extends well beyond its defining symptoms. Psychiatric comorbidity is common across the lifespan [4,5], while adult ADHD is also associated with physical multimorbidity [6], sleep problems [7], and increased mortality risk [8]. Treatment research further suggests that improvement in core symptoms does not necessarily translate into equivalent gains in broader outcomes, including quality of life [9]. These problems are not peripheral to ADHD simply because they fall outside its diagnostic criteria: they may shape diagnostic interpretation, treatment choice, monitoring, prognosis, and day-to-day functioning.

The contributions to this Special Issue approach this broader clinical field from different angles, spanning assessment and informant discrepancies, addiction-related treatment context, menstrual-cycle-related variation, patterns of medication use, sleep and circadian factors, and educationally relevant cognitive function [10,11,12,13,14,15]. Read together, they do not propose another catalogue of problems to be appended to ADHD. Rather, they show that the clinical meaning of the disorder depends partly on the conditions, contexts, and functional demands surrounding its defining symptoms. Some of these observations already bear directly on assessment or treatment; others remain primarily explanatory and may acquire greater clinical relevance as the evidence develops.

The task, then, is neither to make ADHD an explanation for every difficulty nor to reduce it to the symptoms required for diagnosis. Clinical precision requires both: a clear definition of the disorder and attention to the wider problems that shape a patient’s health, functioning, and treatment. In that sense, bridging theory and clinical application means moving beyond the diagnostic core without losing sight of it. We hope this Special Issue invites readers to do precisely that.
